# Individualized rTMS Intervention Targeting Sleep Deprivation‐Induced Vigilance Decline: Task fMRI‐Guided Approach

**DOI:** 10.1111/cns.70087

**Published:** 2024-11-13

**Authors:** Yuanqiang Zhu, Chen Wang, Ziliang Xu, Fan Guo, Yingjuan Chang, Jiali Liu, WenMing Liu, Peng Fang, Minwen Zheng

**Affiliations:** ^1^ Department of Radiology, Xijing Hospital Air Force Medical University Xi'an Shaanxi China; ^2^ Department of Psychiatry, Xijing Hospital Air Force Medical University Xi'an Shaanxi China; ^3^ Department of Military Medical Psychology Air Force Medical University Xi'an Shaanxi China; ^4^ Shaanxi Provincial Key Laboratory of Bioelectromagnetic Detection and Intelligent Perception Xi'an Shaanxi China; ^5^ Military Medical Innovation Center, Fourth Military Medical University Xi'an Shaanxi China

**Keywords:** individualized stimulation target, nap, psychomotor vigilance task, repetitive transcranial magnetic stimulation, sleep deprivation

## Abstract

**Study Objectives:**

Sleep deprivation (SD) is prevalent in our increasingly round‐the‐clock society. Optimal countermeasures such as ample recovery sleep are often unfeasible, and brief naps, while helpful, do not fully restore cognitive performance following SD. Thus, we propose that targeted interventions, such as repetitive transcranial magnetic stimulation (rTMS), may enhance cognitive performance recovery post‐SD.

**Methods:**

We recruited 50 participants for two SD experiments. In the first experiment, participants performed a psychomotor vigilance task (PVT) under three conditions: normal sleep (resting wakefulness), after 24 h of SD, and following a subsequent 30‐min nap. We analyzed dynamic changes in PVT outcomes and cerebral responses across conditions to identify the optimal stimulation target. Experiment 2 adopted the same protocol except that, after the nap, 10‐Hz, sham‐controlled, individualized rTMS was administrated. Then, an analysis of variance was conducted to investigate the ability of stimulation to improve the PVT reaction times.

**Results:**

Through task‐related functional magnetic resonance imaging, we identified cerebral responses within the right middle frontal gyrus (MFG) as the optimal stimulation target. Subsequent application of individualized 10‐Hz rTMS over the right MFG attenuated SD‐induced deterioration of vigilance.

**Conclusion:**

Our findings suggest that combining a brief nap with individualized rTMS can significantly aid the recovery of vigilance following SD. This approach, through modulating neural activity within functional brain networks, is a promising strategy to counteract the cognitive effects of SD.

## Graphical abstract

We recruited 50 healthy volunteers for two sleep deprivation experiments. In Experiment 1, each participant underwent a psychomotor vigilance task under three conditions: (1) after normal sleep, (2) after 30 h of sleep deprivation, and (3) after a subsequent 30‐min nap, while undergoing functional magnetic resonance imaging (fMRI). By analyzing the dynamic changes in task performance and brain responses across these conditions, we aimed to identify the optimal stimulation target. We discovered, for the first time, that the brain response in the right middle frontal gyrus exhibited the least recovery after a nap, leading us to select it as the stimulation target. In Experiment 2, using the same protocol (normal sleep, 30 h of sleep deprivation, 30‐min nap), rTMS stimulation was applied after the nap. Subsequently, we found that individualized rTMS attenuated the sustained attention decline induced by sleep deprivation relative to napping alone. Thus, nap combined with personalized rTMS may facilitate the recovery of sustained attention following sleep deprivation by modulating neural activity in brain functional networks.

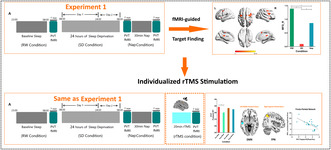

**Abbreviations**
ANOVA: analysis of variances
DARTEL: Diffeomorphic Anatomical Registration through Exponentiated Lie Algebra
DMN: default mode network
DPABI: Data Processing and Analysis for Brain Imaging
FDR: false discovery rate
FPN: frontoparietal network
HF: high‐frequency
ICA: independent component analysis
MFG: middle frontal gyrus
MNI: Montreal Neurological Institute
PVT: psychomotor vigilance task
REST: Resting State fMRI Data Analysis Toolkit
RT: reaction time
rTMS: repetitive transcranial magnetic stimulation
RW: resting wakefulness
SD: sleep deprivation
SPM: statistical parametric mapping
SwE: sandwich estimator

## 1 Introduction

Insufficient sleep is a pervasive issue in contemporary society, impacting not only specialized environments, such as military operations but also various sectors of public life [1, 2, 3]. Studies suggest that a significant proportion of the global adult population sleeps less than the recommended 7–9 h per night, with potential detrimental effects on health, cognition, and performance [4, 5]. Sleep deprivation (SD) is an escalating concern in various professions requiring round‐the‐clock functioning, including healthcare, transportation, and emergency services [6, 7]. SD has been extensively demonstrated to compromise performance across numerous cognitive domains, such as inducing lapses in sustained attention, reducing short‐term memory capacity, and impairing decision‐making skills [8, 9]. These deficits can have serious ramifications, especially for individuals in high‐risk occupations necessitating continuous alertness and precision. Consequently, the effects of SD extend beyond individual well‐being, potentially influencing overall operational efficacy and public safety.

Undoubtedly, ample recovery sleep is the optimal strategy to counteract the effects of SD [10, 11]. Yet, in many sectors of modern society, finding sufficient time for restful sleep is challenging because of professional obligations, shift work, and fast‐paced lifestyles. Therefore, a brief (30‐min) nap is frequently used to alleviate the adverse effects of SD on cognitive function. Naps offer various benefits such as reduced fatigue, increased alertness, and improved performance, including better memory and reaction times (RTs) [12, 13]. However, a short nap is not sufficient to fully restore cognitive performance following SD [14, 15]; thus, rapid, targeted interventions after a brief nap, such as noninvasive brain stimulation techniques, might better restore performance following SD.

The study of noninvasive techniques for modifying cognitive function, such as repetitive transcranial magnetic stimulation (rTMS), has grown exponentially in the past 20 years [16, 17]. In rTMS, an electrical current is produced in the brain cortex by applying a high‐intensity magnetic field. Depending on the stimulation parameters, in particular the frequency of stimulation, excitability in the targeted cortical region can be enhanced (at high frequency [5–20 Hz]) or suppressed (at low frequency [≤ 1 Hz]). High‐frequency (HF) rTMS has had encouraging effects on the cognitive performance of patients with Alzheimer's disease [18] and has been applied to successfully treat cognitive deficits in various neuropsychiatric disorders, including depression [19], schizophrenia [20], and Parkinson's disease [21]. Therefore, by increasing cortical excitability, HF rTMS after a nap might help offset the cognitive performance deterioration induced by SD.

However, as well as the impact of the frequency on the effects of stimulation, the importance of the stimulation site must also be considered. Even subtle changes in the stimulation target might lead to large changes in the effects of rTMS [22]. Hence, determining the optimal rTMS target area is crucial for maximizing performance improvements. Our previous studies adopting functional magnetic resonance imaging (fMRI) demonstrated that frontal and parietal regions are preferentially affected by SD [23, 24], with brain activities within the bilateral middle frontal gyrus (MFG), inferior frontal gyrus, superior parietal gyrus, and supplementary motor area being compromised after SD. Hence, these brain regions are promising candidate stimulation targets to combat cognitive decrements. However, “abnormal” activity within some of these areas might normalize after a brief nap; therefore, targeting the brain area that shows the least “normalization” after a nap might maximize the benefits of rTMS.

In this study, a psychomotor vigilance task (PVT) was used to probe sustained attention, which is one of the cognitive functions most affected by SD [25]. To test our hypothesis, 50 healthy participants were recruited to two SD experiments. During experiment 1, each participant performed a PVT task in a magnetic resonance scanner after resting wakefulness (RW), 24 h of SD, and a subsequent 30‐min nap. Dynamic changes in cerebral responses across the three conditions were analyzed to determine the optimal simulation target. The obtained candidate brain region was then transformed into an individual space to obtain the individualized stimulation target zone. In experiment 2, the same protocol (i.e., the same three conditions) was adopted. After the 30‐min nap, 10‐Hz, randomized, sham‐controlled, and individualized rTMS was administrated. We believe that rTMS, when applied in conjunction with napping, can significantly improve sustained attention.

## 2 Methods

This study was conducted according to the Declaration of Helsinki and approved by the Ethics Committee of Xijing Hospital. Each participant provided written informed consent; all participants were cadets at Air Force Military Medical University. The inclusion criteria were as follows: (1) right handed and (2) aged 18–30 years. The exclusion criteria were (1) the presence or history of a medical disease, (2) the presence or history of sleep disorders, (3) the presence or history of psychiatric disease, (4) being a shift worker, (5) a history of substance abuse or dependence, and (6) a contraindication to magnetic resonance imaging (MRI).

### 2.1 Experiment 1: Procedure

**FIGURE 1:**
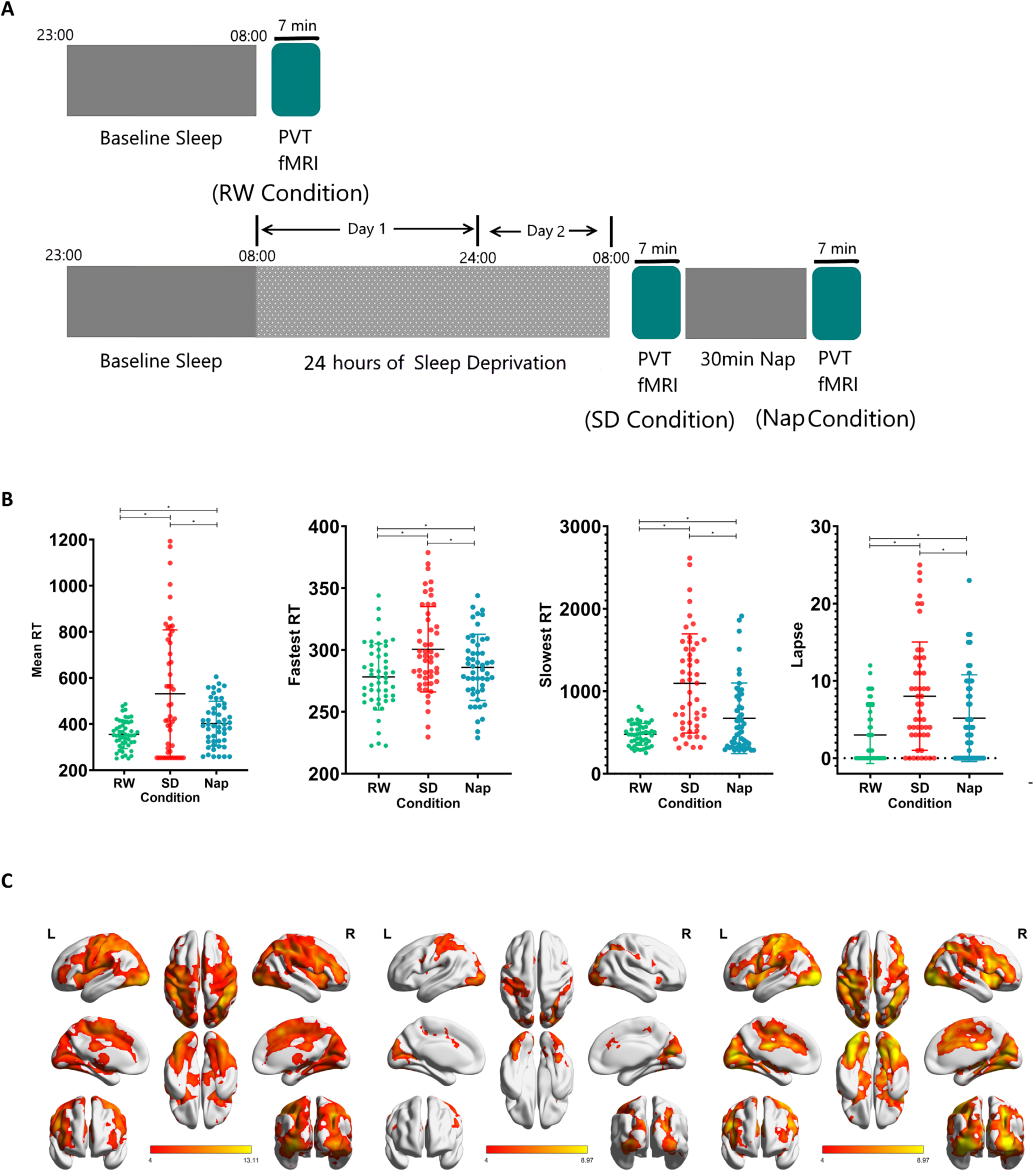
Experiment 1. (A) Participant performing a PVT task in the MR scanner after RW, 24 h of SD, and a subsequent 30 min nap. (B) Significant time effects (*p* < 0.001) were observed for mean RT, fastest RTs, slowest RTs, and number of lapses. Post hoc analysis showed significant pairwise differences between RW, SD, and nap (*p* < 0.01). (C) One‐sample *t*‐test results for cerebral responses during the fastest RT in each condition, *p < 0.05.

All the participants made three visits to the laboratory. During the first visit, they were briefed about the experimental protocol and provided with an Actiwatch (Philips Respironics, Mini Mitter), which was worn on the wrist to record sleep patterns. All participants signed an informed consent form. At the second and third visits, the 24 h of SD was completed, followed by an MRI scan, or an MRI scan was performed after normal sleep. These latter two visits were completed in a pseudo‐random order to minimize the effect of the scanning sequence. To avoid persistent effects of SD, the interval between the last two visits was at least 1 week. The SD process began at 8:00 AM and ended at 8:00 AM on the following day. During SD, participants were allowed to engage in low‐stimulation activities such as reading, watching TV, and surfing the Internet. To minimize the potential for confounding, strenuous physical activities were prohibited, and participants were instructed to avoid consuming any beverages containing caffeine throughout the experiment. These controls were implemented to ensure that any observed effects could be more confidently attributed to the SD and rTMS interventions, rather than to external factors. The temperature was maintained at approximately 23°C, with standard light conditions (340 lx). Two researchers accompanied each participant to prevent the participant from falling asleep. The MRI scans were scheduled between 8:00 AM and 9:00 AM in both the RW and SD conditions. After completing the MRI scans in the SD condition, participants were allowed to sleep for 30 min, during which time polysomnography data were acquired from six electroencephalography (EEG) channels (F3, F4, C3, C4, O1, and O2 [International 10–20 system]) and two electrooculography (EOG) channels (EOG1 and EOG2) referenced to the contralateral mastoids (A1 and A2, respectively). Bipolar submental electromyography (EMG) measures were also obtained. Impedance was kept below 5 kΩ for EEG electrodes and below 10 kΩ for EOG and EMG electrodes. After the 30‐min nap, participants were woken for the final magnetic resonance scan, before which they were given a 20‐min break to clean up; this break minimized possible effects of sleep inertia. The detailed experimental procedure is illustrated in Figure 1A.

### 2.2 Experiment 1: PVT

The ability to sustain attention was measured using a well‐defined PVT described in detail elsewhere [26]. Briefly, participants viewed a computer monitor and were asked to press the space bar on a keyboard to stop a running counter as quickly as possible. Specifically, at random intervals, a counter having millisecond precision began to scroll, and participants were asked to press the space bar to stop the running counter as quickly as possible. After pressing the button, the counter displayed the achieved RT for 1 s as feedback for monitoring task performance. The duration of the task was 7 min, and the inter‐stimulus intervals varied randomly from 2 to 10 s. Performance outcomes of the PVT included the mean RT, mean of the fastest 20% of RTs, mean of the slowest 20% of RTs, and number of attention lapses (RT > 500 ms). The times at which each participant made the fastest 20% of reactions were noted and used as the event regressor in later task‐fMRI analyses.

### 2.3 Experiment 1: MRI Data Acquisition

Imaging data were collected using a 3‐Tesla MRI system (EXCITE; General Electric, Milwaukee, WI, USA) at the Department of Radiology of Xijing Hospital (Air Force Military Medical University, Xi'an, China). A standard birdcage head coil was used, along with restraining foam pads, to minimize head motion and reduce scanner noise. Participants were asked to complete the PVT task in the MRI scanner, and stimuli were projected onto a screen using a liquid‐crystal display projector viewed by participants through a mirror positioned above them on the head coil. Parameters of the task‐fMRI imaging included a gradient echo‐planar imaging sequence of 210 images, an echo time of 30 ms, a repetition time of 2 s, a 64 × 64 data matrix, 33 slices, and a field of view of 240 × 240 mm. In the RW condition, high‐resolution, T1‐weighted, three‐dimensional anatomical data were also acquired using a three‐dimensional magnetization‐prepared rapid gradient‐echo sequence with a repetition time of 8.2 ms, echo time of 3.18 ms, field of view of 256 × 256 mm, 256 × 256 data matrix, flip angle of 9°, thickness of 1 mm, and 196 slices with no gaps.

### 2.4 Experiment 1: MRI Data Preprocessing

The fMRI images were preprocessed using Data Processing and Analysis for Brain Imaging (http://rfmri.org/dpabi) software, which synthesizes procedures in the Resting State fMRI Data Analysis Toolkit (http://www.restfmri.net) and Statistical Parametric Mapping (SPM12; www.fil.ion.ucl.ac.uk/spm). The first 10 images were removed to ensure magnetization equilibrium, and the remaining 200 images were subjected to slice‐time correction and realigned to correct for head motion, in which the mean framewise displacement was calculated. Participants with maximal translation exceeding 2 mm or maximal rotation of 2° were excluded. The Friston‐24 model was then used to regress both head motion effects and nuisance signals from cerebrospinal fluid and white matter. Subsequently, the fMRI data were normalized to Montreal Neurological Institute (MNI) space using the diffeomorphic anatomical registration through the exponentiated lie algebra method. Finally, the resulting images were smoothed with a Gaussian kernel having a full width at half maximum of 6 mm and then bandpass filtered (0.01–0.08 Hz).

### 2.5 Experiment 1: MRI Data Processing

Task‐related brain activation data were processed using a two‐level mixed‐effects model [23]. In the first level, for each participant, the fastest 20% of responses for PVT events were modeled as delta (stick) functions, convolved with the hemodynamic response function, and analyzed using a general linear model. The six head motion parameters obtained during realignment were applied to take all head motion into account, and a high‐pass filter with a 128‐s cutoff was used to remove effects related to low‐frequency changes, such as the beating of the heart. In this way, we were able to identify brain regions that were significantly more active or inactive during the fastest events than during typical events. Parameter estimates obtained for the predictor (the fastest 20% of events) for each participant were then input to the second level using the sandwich estimator (SwE), to account for the within‐subject correlations in our longitudinal data [27].

There were several steps in the second level. First, for each imaging condition (RW, 24 h of SD, and a 30‐min nap), a one‐sample *t*‐contrast was specified in the SwE contrast manager to examine the overall PVT activation patterns. Second, an *F*‐contrast was specified in the SwE contrast manager to determine whether there were significant differences in PVT activation patterns across conditions. Additionally, post hoc “*t* comparison images” of sessions were generated in the SwE contrast manager. Finally, estimates of brain activation in brain regions that showed significant differences across the three conditions were extracted and plotted for visualization.

### 2.6 Experiment 1: Statistical Analysis

Before performing parametric tests, the data distributions for each measure (mean RT, fastest RT, slowest RT, and number of lapses) across the three conditions (RW, SD, Nap) were assessed for normality using the Shapiro–Wilk test. The results indicated that the data were normally distributed (*p* > 0.05 for all measures and conditions), justifying the subsequent use of repeated‐measures analysis of variance (ANOVA). For the PVT outcomes (mean RT, mean of the fastest RTs, mean of the slowest RTs, and mean number of PVT lapses), one‐way repeated‐measures ANOVA was carried out with an alpha of 0.01 to account for multiple comparisons. In post hoc comparisons, the alpha was set at 0.05 with Bonferroni correction. Correcting for multiple comparisons in the fMRI data analysis was accomplished using the false discovery rate (FDR) (Guillaume et al., 2014). For the fastest PVT activation under different conditions, “*F* comparisons” across different conditions, and post hoc “*t* comparisons” between conditions, the statistical threshold was set as positive FDR (pFDR) < 0.05. This setting corrected for multiple comparisons across the whole brain.

### 2.7 Experiment 2: Study Procedure

The procedure for experiment 2 was the same as that for experiment 1, except that rTMS was implemented after the 30‐min nap. After rTMS, participants were scanned again. In total, the participants were scanned four times (RW, SD, nap, and rTMS). The PVT task, the task‐fMRI scanning parameters, and the data‐preprocessing steps in experiment 2 were identical to those in experiment 1. The interval between experiments 1 and 2 was set at 2 months to allow statistical analysis of the results of experiment 1. The detailed experimental procedure is illustrated in Figure 3A.

### 2.8 Experiment 2: rTMS Setup and Localization

Before setting up the rTMS, participants were randomized to receive either active or sham rTMS. One‐way repeated‐measures ANOVA and post hoc analysis of the results of experiment 1 identified the right MFG as the candidate stimulation target (see the Results section). Participants received rTMS modulation over the right MFG according to the following protocol. Each rTMS session involved the delivery of 30 trains of TMS pulses at 10 Hz for 10 s (100 pulses/train) with a 20‐s intertrain interval, resulting in 3000 pulses per session over a total of 15 min [28, 29]. The stimulation intensity was set at 80% of the resting motor threshold, which was measured as the minimum intensity of stimulation evoking an electromyographic response of 550 mV on the first dorsal interosseus muscle of the hand contralateral to the stimulated hemisphere in at least 5 of 10 trials [30]. For individualized stimulation, the MNI coordinates (30, 0, 48) were adjusted using the inverse normalization method embedded in SPM12 (i.e., normalization based on the inverse deformation field). All rTMS sessions were assisted by a neuronavigation system. Once the location of the stimulation target was determined, the coordinates were included in the neuronavigation system. The integration of the neuronavigation unit into the system guaranteed continuous and precise targeting.

In the control condition, sham rTMS was administered to mimic the sensory experience of active rTMS with no therapeutic effect. The sham rTMS setup involved positioning the rTMS coil at the same location as in the active treatment, but it was tilted away from the scalp at a 90° angle. This orientation prevented the magnetic field from effectively penetrating the skull and stimulating the brain tissue. To ensure that participants were blinded to the treatment condition, the sham procedure was designed to replicate the auditory and tactile sensations of the active rTMS. The coil clicks and the sensation of coil contact with the scalp were identical to those in the active rTMS sessions. This careful replication of sensory cues was intended to maintain the credibility of the sham condition, that is, to ensure that participants were unaware of whether they were receiving real or sham stimulation [29, 31]. By closely mimicking the active rTMS procedure, the sham rTMS condition served as a robust control, allowing us to isolate the specific effects of the active rTMS on cognitive function and neural activity. To investigate the stimulation effect for the fastest PVT RTs, a mixed two‐way repeated‐measures ANOVA was conducted with a group (real rTMS/sham rTMS) as the between‐subjects variable and condition (pre‐stimulation/after stimulation) as the within‐subjects variable.

### 2.9 Experiment 2: Independent Component Analysis (ICA)

Previous studies indicated that the benefits of rTMS on cognition might be mediated by distributed functional networks [32]. We therefore investigated how single rTMS stimulation of the MFG affects intrinsic within‐network functional connectivity. Group spatial ICA and functional network connectivity analysis were performed using GIFT software (University of New Mexico, Albuquerque, NM, USA) [33]. The optimal number of components, estimated using the minimum description length criterion, was set at 25. After data reduction via principal component analysis, ICA decomposition was applied to the concatenated data using the extended infomax algorithm. Independent components were then back‐reconstructed for each participant, and participants were finally characterized using the 25 independent components. The degree to which a voxel belongs to a network is represented by the voxel's value. As stated in our previous paper [24], the default mode network (DMN) and frontoparietal network (FPN) are involved in the modulation of sustained attention, and we therefore identified two components corresponding to these two functional networks using a widely applied visual inspection procedure (3, 6).

For each participant, the two components were extracted and scaled to spatial z‐maps. For each network (i.e., the DMN and FPN), mixed two‐way repeated‐measures ANOVA was conducted with a group (real rTMS/sham rTMS) as the between‐subjects variable and condition (pre‐stimulation/after stimulation) as the within‐subjects variable. The level of significance was set at *p* < 0.05, corrected for multiple comparisons using the FDR method. In addition, we tested planned condition comparisons (pre‐stimulation vs. post‐stimulation) for each group. The statistical threshold was set as pFDR < 0.05.

## 3 Results

A total of 50 participants successfully completed the two experiments. Detailed demographics are listed in Table 1. Participants did not smoke or consume stimulants during the SD sessions.

**TABLE 1:** Demographic characteristics, objective sleep measures and PVT performances.

Gender (male/female)	27/23
Age (years)	23.08 ± 1.65
Body mass index	22.90 ± 0.78
Objective sleep characteristics from Actiwatch	
Number of wakening each night	27.65 ± 6.08
Sleep duration all night	6.76 ± 1.48
Sleep durations before workdays	6.38 ± 1.36
Sleep durations before free days	7.06 ± 1.49
Sleep efficiency in %	85.10 ± 4.65
Sleep latency in minutes	15.27 ± 5.92

PVT performances	Experiment 1	Experiment 2	Statistical value	*p*
RW condition				
Mean RT	355.16 ± 60.53	356.69 ± 64.55	−0.11^a^	0.91
Fastest RT	280.64 ± 22.71	281.86 ± 24.12	−0.26^a^	0.79
Slowest RT	476.44 ± 132.39	480.63 ± 108.51	−0.18^a^	0.85
Number of lapse	3.00 ± 3.66	2.88 ± 3.11	0.22^a^	0.82
SD condition				
Mean RT	531.52 ± 273.88	530.45 ± 257.16	0.02^a^	0.98
Fastest RT	302.14 ± 31.94	302.36 ± 31.59	−0.03^a^	0.97
Slowest RT	1161.43 ± 797.63	1165.61 ± 900.29	−0.02^a^	0.98
Number of lapse	8.02 ± 6.93	8.08 ± 6.71	−0.04^a^	0.96
Nap condition				
Mean RT	402.17 ± 96.05	401.11 ± 130.46	0.04^a^	0.96
Fastest RT	287.25 ± 24.32	286.47 ± 24.95	0.17^a^	0.87
Slowest RT	649.43 ± 439.02	645.01 ± 371.12	0.02^a^	0.96
Number of lapse	5.18 ± 5.55	5.10 ± 7.45	0.03^a^	0.97

*Note:* Normality tests indicated that all data under each condition followed a normal distribution.

^a^ T value obtained by using the paired *t*‐test.

### 3.1 Experiment 1

#### 3.1.1 Behavioral Results

One‐way repeated‐measures ANOVA revealed a significant effect of time on the mean RT (*F*_2,98_ = 14.51, *p* < 0.001), mean of the fastest RTs (*F*_2,98_ = 8.28, *p* < 0.001), mean of the slowest RTs (*F*_2,98_ = 21.49, *p* < 0.001) and number of attention lapses (*F*_2,98_ = 8.30, *p* < 0.001). Post hoc pairwise comparisons revealed significant differences between the RW, SD, and nap conditions. Figure 1B shows the significantly reduced RT under the nap versus SD condition. Meanwhile, the RT under the nap condition remained significantly longer than that under the RW condition, suggesting that recovery was insufficient.

#### 3.1.2 Imaging Results: PVT Activations Under Different Conditions

A one‐sample *t*‐test was conducted to investigate the cerebral response for the fastest RT under each condition. Figure 1C shows that the activation pattern under the RW condition was similar to that observed in our previous studies; that is, the regions of brain activation mainly belonged to the FPN, dorsal and ventral attention network, and visual and salience networks. The activation pattern under the nap condition was generally similar to that under the RW condition, except that the activation strength and extent were compromised. Meanwhile, a weaker activation pattern was observed for the SD condition, suggesting task disengagement in several brain regions after SD.

#### 3.1.3 Imaging Results: ANOVA of PVT Activation

**FIGURE 2:**
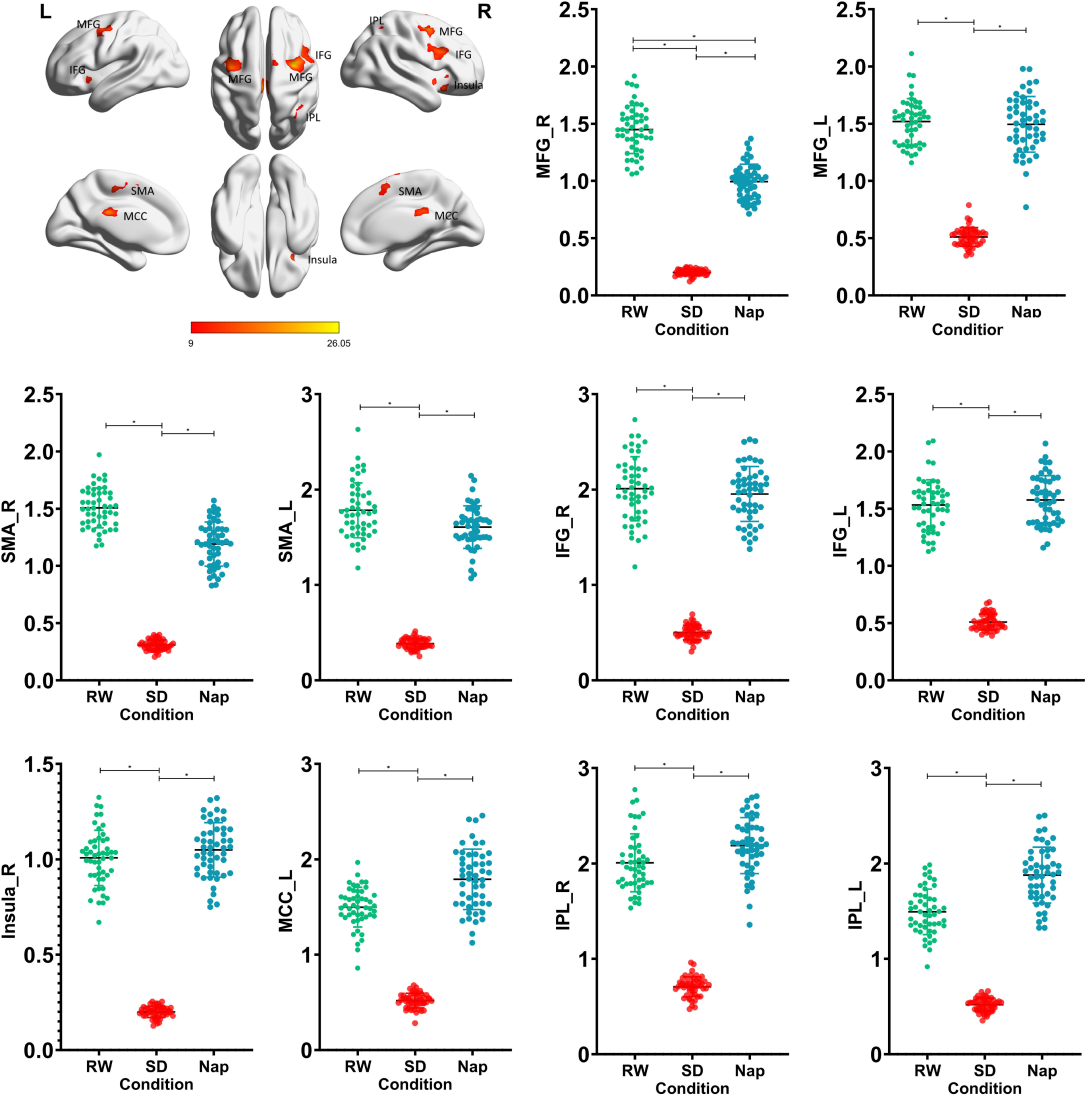
Significant changes were observed in multiple brain regions, including MFG, SMA, IFG, insula, MCC, and IPL. Notably, only the pairwise differences in the right MFG mirrored the differences in the fastest PVT RT, with significant differences across all conditions. Differences in other regions were primarily observed between SD and the other two conditions. IFG, inferior frontal gyrus; IPL, inferior parietal lobule; L, left; MCC, middle cingulate cortex; MFG, middle frontal gyrus; R, right; SMA, supplementary motor area, *p < 0.05.

One‐way repeated‐measures ANOVA was adopted to investigate dynamic changes in PVT activation across the three conditions. Figure 2 and Table 2 show the statistically significant changes in cerebral responses in the right MFG, left MFG, right supplementary motor area, left supplementary motor area, right inferior frontal gyrus, left inferior frontal gyrus, right insula, left middle cingulate cortex, right inferior superior parietal gyrus, and left inferior superior parietal gyrus. The individual parameter estimates (individual activations) of these brain regions were extracted across the three conditions, and post hoc *t*‐tests were conducted to check for pairwise differences between conditions. Figure 2 reveals that the pairwise differences in the right MFG were similar to those observed for the fastest PVT RTs, with significant differences seen between the RW and SD conditions, RW and nap conditions, and SD and nap conditions. Meanwhile, the differences in other regions were mainly caused by differences between the SD condition and the other two conditions. Therefore, the right MFG (30, 0, 48) was selected as the candidate stimulation target.

**TABLE 2:** Peak coordinates of significant brain regions in ANOVA results for the activation of fastest PVT RT.

Regions‐(ANOVA)	Number of voxels	Peak coordinates (MNI)			*F*‐value
		*x*	*y*	*z*	
Middle frontal gyrus					
Right	269	30	0	48	26.04
Left	221	−39	−6	54	17.09
Supplemental motor area					
Right	136	6	12	54	13.60
Left	75	−9	−21	51	13.65
Inferior frontal gyrus					
Right	278	48	15	27	19.52
Left	80	−39	15	−9	12.71
Insula					
Right	185	33	18	−18	19.17
Middle cingulate cortex					
Left	157	−3	−27	24	18.93
Inferior parietal gyrus					
Right	63	30	−54	54	12.10
Left	42	−30	−57	42	13.13

*Note:* Significance was set at *p* < 0.05 (false discovery rate corrected).

### 3.2 Experiment 2

#### 3.2.1 Behavioral Results

**FIGURE 3:**
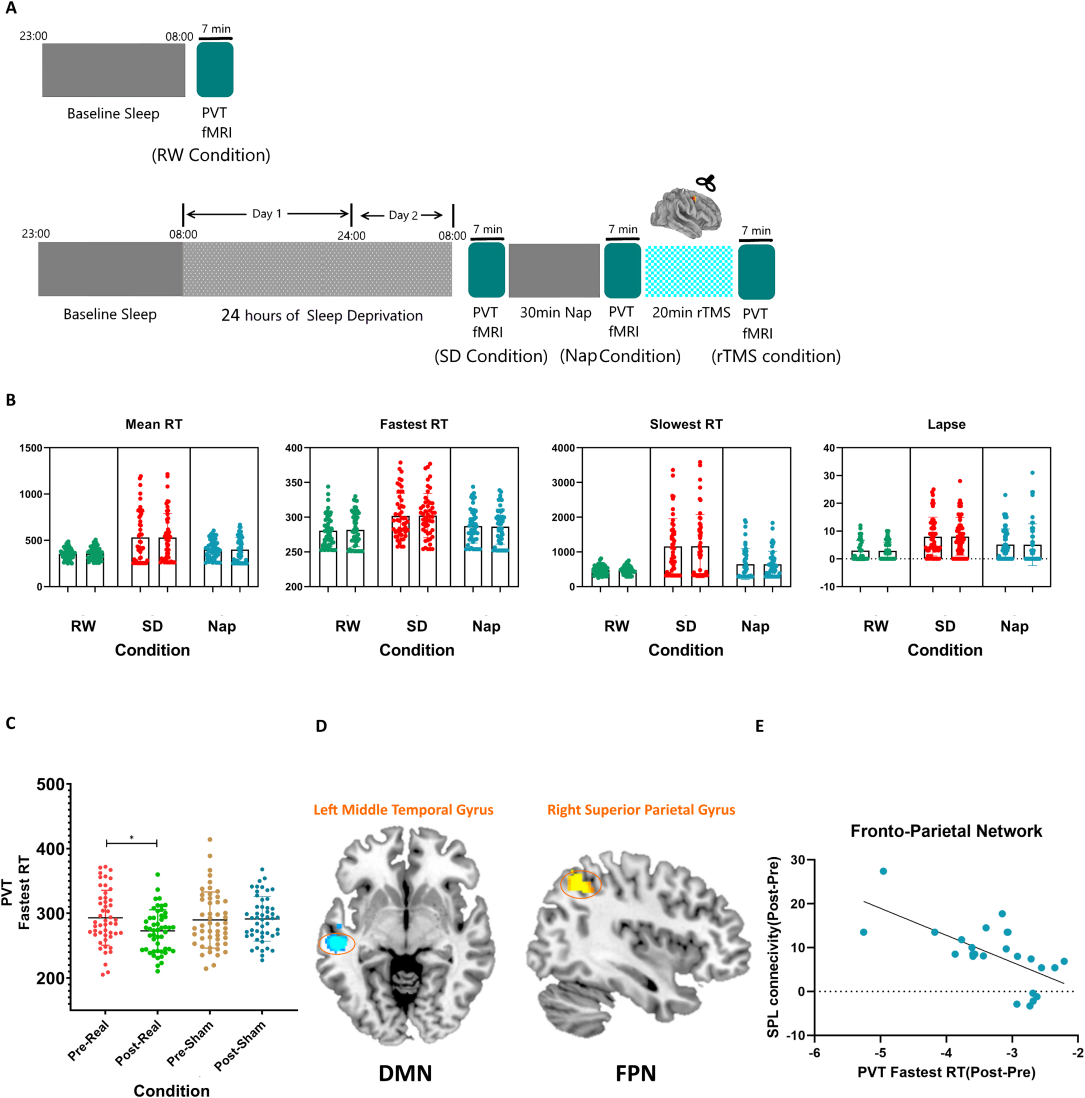
Experiment 2. (A) Participants performed a PVT task in the MR scanner after resting wakefulness (RW), 24 h of sleep deprivation (SD), a 30‐min nap, and 20‐min repetitive transcranial magnetic stimulation (rTMS). (B) No differences in PVT outcomes were found between Experiments 1 and 2 for each condition (RW, SD, nap). (C) Mixed two‐way repeated‐measures ANOVA revealed a significant main effect of condition and a significant interaction effect. Reaction time was significantly reduced after rTMS, with real rTMS showing the greatest improvement in processing speed. (D) For participants receiving real rTMS, planned comparisons (pre‐stimulation vs. post‐stimulation) revealed increased functional connectivity in the frontoparietal network (FPN), primarily at the right superior parietal gyrus, and reduced functional connectivity within the default mode network (DMN), primarily at the medial frontal gyrus. (E) Increased functional connectivity in the right superior parietal gyrus after stimulation was negatively correlated with a reduction in the fastest reaction time (RT) for participants receiving real rTMS, *p < 0.05.

Table 1 and Figure 3B show that there were no differences in PVT outcomes between experiments 1 and 2. As in previous studies, task practice effects in the PVT were minimal in the current study, and all participants were instructed to perform the PVT at least five times to prevent potential practice effects. The results indicate that the three conditions were nearly identical in the two experiments, guaranteeing the next rTMS stimulations.

Mixed two‐way repeated‐measures ANOVA revealed a significant main effect of condition (*F*_1,24_ = 8.82, *p* < 0.01) and a significant interaction effect (*F*_1,24_ = 145.17, *p* < 0.001). As shown in Figure 3C, the RT was significantly reduced after rTMS, and the real rTMS improved the processing speed the most.

#### 3.2.2 Imaging Results

We selected the DMN and FPN from the group ICA for further analysis. However, mixed two‐way repeated‐measures ANOVA indicated no significant effects. The planned condition comparisons (pre‐stimulation vs. post‐stimulation) revealed no significant differences in the sham group, but in the real group, FPN connectivity was increased, mainly in the right superior parietal gyrus, and DMN connectivity was reduced, mainly in the medial frontal gyrus (Figure 3C and Table 3). Interestingly, in the real rTMS group, the increased functional connectivity in the right superior parietal gyrus seen after stimulation was negatively correlated with the changes in the fastest RT.

**TABLE 3:** Peak coordinates of significant brain regions after Real rTMS administration.

Regions (Post‐Pre)	Number of voxels	Peak coordinates (MNI)			*t*‐value
		*x*	*y*	*z*	
Default mode network					
Left middle temporal gyrus	87	−54	−22	−6	−14.88
Frontal parietal network					
Right superior parietal gyrus	134	38	−56	44	22.11

*Note:* Significance was set at *p* < 0.05 (false discovery rate corrected).

## 4 Discussion

Our pilot study validated the effectiveness of rTMS after a brief nap for improving sustained attention in the context of SD. Investigation of the dynamic changes in PVT task‐related cerebral responses across the three conditions showed that the MFG recovered least well after the nap and was thus selected as the stimulation target. By modulating functional connectivity within the FPN and DMN, individualized 10‐Hz rTMS showed promise for improving the vigilance of sleep‐deprived individuals.

The effects of SD on cognitive performance have been well documented in the literature [8, 34]. Converging evidence indicates that the most reliable neurobehavioral change induced by SD is an impairment of sustained attention [35], which forms the bedrock of other, more complex components of cognition. Many cognitive performance deficits induced by SD are attributable largely to the impaired capacity to sustain a sufficient level of vigilance [36]. In several professional fields where round‐the‐clock service is expected, such as healthcare, transportation, and emergency services, opportunities for quality sleep are often scarce. Consequently, a reduction in sustained attention can impact critical aspects of job performance, including decision making, planning, and leadership. This could potentially increase the risk of errors and accidents, affecting not just individual performance but also public safety. In the current study, SD adversely affected the mean RT, fastest RT, slowest RT and number of attention lapses, suggesting that SD causes an overall slowing of reactions, decreases alertness, and increases errors of omission and cognitive instability [37]. A brief nap improved the previously degraded neurobehavioral performance. However, PVT impairments were only partially reversed, consistent with previous studies showing that a brief nap restores nearly 50% of the cognitive decrement [14, 15].

Caffeine and energy drinks have been used as countermeasures against the cognitive consequences of SD [38, 39]. According to previous works [40, 41], when countermeasures against sleepiness are combined with a brief nap, the benefits to performance can be greater than those of the individual treatments alone. Consistent with this, our study found that rTMS after a nap was adequate for recovery from prolonged sleep loss. The optimal alertness level, as indicated by the fastest PVT RTs, after real rTMS (post‐nap) was nearly identical (*p* = 0.2) to that in the RW condition. Notably, neurobehavioral deficits arising from SD were significantly different among, but stable within, individuals [42]. The inter‐individual differences in variability in cognitive deficits were trait‐like and stable, irrespective of sleep history [43]. We recently suggested that functional network properties and white matter integrity can be used to predict trait‐like vulnerability [25, 44]. Importantly, previous studies found that PVT performance is reproducible across repeated exposures to SD (even when separated by an average of 7 months) [45, 46]. Consistent with these findings, the performance in our RW condition was nearly identical between experiments 1 and 2 (*p* > 0.5). Considering the short interval between the two experiments and the completion of more than five PVT practice runs before each experiment, the high reproducibility of PVT performance in the current study was reasonable. As well as the behavioral results, cerebral activation in task‐fMRI analyses was also reproducible [47]. We were thus able to conduct two SD experiments to verify the feasibility of using noninvasive brain stimulation techniques to enhance performance restoration after SD.

Dynamic changes across the RW, SD, and nap conditions were mainly observed in the MFG, inferior parietal gyrus, medial frontal gyrus, and supplementary motor area. The MFG, inferior parietal gyrus, and supplementary motor area are core regions of the FPN [48]. Our post hoc analysis indicated that the cerebral responses within these regions were strongest under the RW condition, suggesting top‐down control of sustained attention by the FPN. Involvement of prefrontal and parietal lobes has consistently been observed in neuroimaging studies [23, 36]. Our previous study, involving repeated fMRI scanning during SD, found significant dynamic changes in cortical activation in response to the fastest PVT in the MFG, inferior frontal gyrus, superior parietal gyrus, and paracentral lobule [23]. An earlier study by Drummond and colleagues found cortical activation in response to the fastest PVT in higher‐order brain regions encompassing frontal and parietal regions [26]. Additionally, our recent study found that fractional anisotropy of the superior longitudinal fasciculus contributed greatly to the classification of PVT‐defined SD‐vulnerable and SD‐resistant participants [25]; the superior longitudinal fasciculus is an extensive longitudinal white‐matter tract connecting the frontal and parietal lobes. All these findings show the importance of the FPN in regulating sustained attention. Meanwhile, for core regions of the DMN, such as the inferior frontal gyrus, a different activation pattern was found, and post hoc analysis indicated that the cerebral response was strongest under the nap condition. The DMN is a brain network that is typically deactivated when performing cognitive tasks. Less deactivation (greater activation) of the DMN has been extensively reported to be associated with poor behavior, such as delayed attention and lapses in attention [49, 50]. Therefore, the dynamic changes observed in the inferior frontal gyrus suggest decreased efficiency of the DMN in terms of segregation from the PVT task.

The activation pattern observed in the right MFG in this study was similar to the pattern of changes in PVT outcomes: the strongest activation (best performance) in the PVT occurred during the RW condition, with the weakest activation (worst performance) seen during the SD condition; the nap condition fell between these two conditions. Although the activation (PVT performance) recovered partially, there were significant differences between the other two conditions. The right MFG was thus selected as the stimulation target. It has previously been found that the MFG serves as a connector hub linking the FPN and DMN, playing important roles in information integration across different brain networks [51]. It is not surprising that, compared with the pre‐stimulation (nap) condition, the administration of real rTMS in our study resulted in increased functional connectivity in the FPN and decreased functional connectivity in the DMN. We believe that rTMS could enhance the advantages of napping, contributing to improved sustained attention. Our previous study revealed that anti‐correlation between the DMN and FPN functionally maintained performance during the worst period of SD [24], and another study found that competition between the “task‐negative” DMN and “task‐positive” FPN mediates attention processes [52]. Two studies have investigated the ability of rTMS over the left dorsolateral prefrontal cortex (DLPFC) to improve cognitive performance after SD [53, 54]; however, contradictory results were obtained, possibly resulting from the arbitrary choice of target. Because the DLPFC comprises multiple distinct anatomical subregions [55], it is a common target in several diseases, such as depression, insomnia, and schizophrenia, as well as for symptoms such as pain. However, stimulation of the DLPFC after SD might lack specificity. In the present study, the target was obtained using task‐fMRI, and placement of the rTMS coil was assisted by robot‐guided neuronavigation; this optimized contact between the coil and scalp and compensated for any head movement. These techniques helped determine the optimal stimulation target and ensured proper and reproducible positioning of the coil. Finally, combined with a brief nap, individualized, fMRI‐guided 10‐Hz rTMS improved sustained attention after SD.

The primary limitation of this study is the homogeneity of the participants, all of whom were cadets from the Air Force Military Medical University. This may limit the generalizability of the findings to non‐military populations. The demanding nature of the SD task necessitated the use of this specific group. However, future studies should include a more diverse participant pool to validate the findings across different groups. This limitation should be considered when interpreting the results. Another limitation is the lack of long‐term follow‐up assessments. Our study focused on immediate post‐treatment outcomes following the combined nap and rTMS intervention. As a result, the durability of the cognitive improvements over time remains unclear. Future research should include follow‐up assessments to evaluate the longevity of the observed effects, and to better understand the practical utility of this intervention in real‐world settings.

## 5 Conclusion

On the basis of the behavioral and task‐related fMRI results in the first experiment, we conclude that the right MFG is the optimal stimulation target for targeted interventions after SD. On the basis of the individualized 10‐Hz rTMS in the second experiment, we conclude that rTMS combined with a brief nap attenuated the sustained deterioration in attention performance. We believe that rTMS will enhance the benefits of napping for sustained attention in military situations by modulating functional connectivity within several functional brain networks.

## Author Contributions

M.Z. and P.F. designed the study. C.W. and Y.C. collected the data. Z.X. analyzed the data. Y.Z., F.G. and W.L. drafted the manuscript. M.Z. and P.F. edited and approved the manuscript.

## Ethics Statement

This investigation was performed in accordance with the Declaration of Helsinki and was approved by the Ethical Committee of the Sports Institute of Xijing Hospital.

## Consent

Consent for publication has been obtained.

## Conflicts of Interest

The authors declare no conflicts of interest.

## Data Availability

The datasets used and/or analyzed during the current study are available from the corresponding author upon reasonable request.
